# Neutral Sphingomyelinase 2 (nSMase2)-dependent Exosomal Transfer of Angiogenic MicroRNAs Regulate Cancer Cell Metastasis*[Fn FN2]

**DOI:** 10.1074/jbc.M112.446831

**Published:** 2013-02-25

**Authors:** Nobuyoshi Kosaka, Haruhisa Iguchi, Keitaro Hagiwara, Yusuke Yoshioka, Fumitaka Takeshita, Takahiro Ochiya

**Affiliations:** From the ‡ Division of Molecular and Cellular Medicine, National Cancer Center Research Institute, 5-1-1, Tsukiji, Chuo-ku, Tokyo 104-0045, Japan,; the § Pharmacology Research Laboratories, Dainippon Sumitomo Pharma Co., Ltd., 1-98 Kasugadenaka 3-chome, Konohana-ku, Osaka 554-0022, Japan, and; the ¶ Department of Biological Information, Graduate School of Bioscience and Biotechnology, Tokyo Institute of Technology, Yokohama, Kanagawa 226-8501, Japan

**Keywords:** Angiogenesis, Cell Biology, Cell-cell Interaction, Exosomes, Metastasis, MicroRNA, Tumor Microenvironment, Extracellular Vesicles, Cell-cell Communication, miR-210

## Abstract

The release of humoral factors between cancer cells and the microenvironmental cells is critical for metastasis; however, the roles of secreted miRNAs in non-cell autonomous cancer progression against microenvironmental cells remain largely unknown. Here, we demonstrate that the neutral sphyngomyelinase 2 (nSMase2) regulates exosomal microRNA (miRNA) secretion and promotes angiogenesis within the tumor microenvironment as well as metastasis. We demonstrate a requirement for nSMase2-mediated cancer cell exosomal miRNAs in the regulation of metastasis through the induction of angiogenesis in inoculated tumors. In addition, miR-210, released by metastatic cancer cells, was shown to transport to endothelial cells and suppress the expression of specific target genes, which resulted in enhanced angiogenesis. These findings suggest that the horizontal transfer of exosomal miRNAs from cancer cells can dictate the microenviromental niche for the benefit of the cancer cell, like “on demand system” for cancer cells.

## Teaser

**Background:** Contribution of exosomal microRNAs to cancer metastasis remains unknown.

**Results:** Exosomal angiogenic microRNAs secreted by metastatic cancer cells promote the metastasis through the activation of endothelial cells.

**Significance:** This is the first to connect cancer metastasis to the exosomal microRNA *in vivo*.

## Introduction

The secretion of humoral factors from cancer cells to microenvironmental cells is essential for metastasis during cancer development (1). Although microRNAs (miRNAs)^3^ are known as tumor suppressors of cell autonomous malignancy phenotypes such as metastasis (2) and multidrug resistancy (3), the roles of miRNAs in non-cell autonomous cancer progression against microenvironmental cells remain largely unknown. The existence of secretory RNA has been known for many years (4, 5), and recent reports have shown that miRNAs (6), which regulate various types of biological phenomena through the regulation of a variety of target genes, are secreted from cells via the exosome (7, 8). These findings have raised the possibility that RNAs, including miRNAs, may serve as novel humoral factors in cell-cell communication (9). We recently demonstrated that miRNAs are released through neutral sphingomyelinase 2 (nSMase2)-regulated secretory machinery and that these secretory miRNAs are transferable and functional in recipient cells (10). Furthermore, we also found that a tumor-suppressive miRNA secreted from non-cancerous cells via this pathway could be transported between cells and exert gene silencing in the recipient cancer cells, thereby leading to an inhibition of cancer cell growth (11). In the last few years, it has become clear that exosomal miRNAs play critical roles in mediating cell-cell communication, specifically between immune cells, endothelial cells and cancer cells (12–17). These findings provide evidence that exosomal miRNAs are required for cell-cell communication in various physiological and pathological conditions, although the contribution of extracellular miRNAs to cancer metastasis remains largely unknown (9). Here, we first demonstrated that horizontal transfer of exosomal miR-210 from metastatic cancer cells could dictate the microenvironmental endothelial cells to the benefit of the cancer cells, which contributed to cancer metastasis. Preventing the expression of nSMase2 in metastatic cancer cells abrogates the metastatic ability of cancer cells to target lung tissues, whereas reconstitution via the administration of exosomes isolated from metastatic cancer cells rescued this phenomenon. In this context, the number of endothelial cells in inoculated tumors was proportional to the expression level of nSMase2 in cancer cells. In fact, exosomes derived from a metastatic cancer cell line enhanced the capillary formation and migration of endothelial cells *in vitro*. Interestingly, the expression profiles of exosomal miRNAs obtained from metastatic cancer cells demonstrated that a set of angiogenic miRNAs were highly concentrated in these exosomes. One of them, miR-210, enhanced the angiogenesis through the suppression of specific target gene, which resulted in enhanced angiogenesis. These results revealed that cancer cells provide nSMase2-regulated exosomal miRNAs to endothelial cells to promote their metastatic initiation efficiency.

## EXPERIMENTAL PROCEDURES

### 

#### 

##### Reagents

Goat polyclonal anti-Alix (Q-19; sc-49268) and donkey anti-goat IgG (HRP; sc-2020) were purchased from Santa Cruz Biotechnology. Mouse monoclonal anti-HSP70, clone 7/HSP70 (610607), and mouse monoclonal ant-human CD63 antibody (556019) were purchased from BD Biosciences. Rabbit polyclonal anti-CD31 antibody (ab28364) was from Abcam. Peroxidase-labeled anti-mouse antibodies were purchased from GE Healthcare (NA931V). GW4869 was purchased from Calbiochem (Darmstadt, Germany). Geneticin and puromycin were purchased from Invitrogen.

##### Cell Culture

4T1 cells, a mouse breast cancer cell line, MCF7, non-metastatic breast cancer cells, and MCF10A, normal mammary epithelial cells, were obtained from the American Type Culture Collection (Manassas, VA). MDA-MB-231-D3H1 and MDA-MB-231-D3H2LN, a metastatic human breast cancer cell line, were obtained from Xenogen. 4T1, MCF7, MDA-MB-231-D3H1, and MDA-MB-231-D3H2LN were cultured in RPMI containing 10% heat-inactivated FBS and antibiotic-antimycotic (Invitrogen) at 37 °C in 5% CO_2_. Human umbilical cord vein endothelial cells (HUVECs) were purchased from Lonza and cultured in EBM-2 BulletKit (Lonza) supplemented with 2% FBS.

##### Exosome Purification

Exosomes were purified by differential centrifugation as described previously (10). The exosome fraction was measured for its protein content using the Micro BCA protein assay kit (Thermo Scientific, Wilmington, DE).

##### Tube Formation Assay

HUVECs (100,000) cells were cultured on 150 μl of Matrigel (Sigma) in culture medium for 16 h in 24-well plate. The degree of tube formation was quantified by measuring the number of branches in five randomly chosen fields from each well using NIH ImageJ software. For rescue experiments, HUVECs were transfected using Dharmafect reagent (Dharmacon) according to the manufacturer's recommendations with anti-control or anti-miR-210 (Ambion). After 24 h of posttransfection, cells were seeded onto Matrigel as described above with 1 μg of exosome.

##### Establishment of Stable Cell Lines

A stable 4T1 and MDA-MB-231-D3H2LN nSMase2-modified cell lines that expressed mouse nSMase2 shRNA, human nSMase2 shRNA, and pCT-CD63-GFP were generated by selection with puromycin. A stable 4T1 and MDA-MB-231-D3H2LN cell lines that overexpresses human nSMase2 were generated by selection with geneticin. 4T1 cells or MDA-MB-231-D3H2LN were transfected with 0.5 μg of the vector at 90% confluency in 24-well dishes using a Lipofectamine LTX reagent in accordance with the manufacturer's instructions.

##### Co-culture Experiments

Well inserts for 24-well plates with a 0.4-μm pore-sized filter were purchased from BD and used following the manufacturer's instructions. 4T1 control cells, 4T1-nSMase2-KD cells, 4T1-siLuc cells, or 4T1-CD63-GFP cells (100, 000) were seeded into the well inserts. HUVECs (200, 000) were seeded into 24-well plates.

##### Confocal Microscopy

Confocal microscopy was done on an Olympus laser scanning microscope FV10i (Olympus). Filters used were 489–510 nm (GFP and Alexa Fluor 488) and 577–603 nm (Alexa Fluor 568).

##### Immunoblot Analysis

Exosomes were lysed in a 2% SDS buffer, and equal amounts of protein were loaded onto an SDS-PAGE gel. Anti-Alix (1:200), anti-HSP70 (1:1,000), and anti-CD63 (1:200) were used as primary antibodies. The dilution ratio of each antibody is indicated in *parentheses*. Two secondary antibodies (peroxidase-labeled anti-goat and anti-mouse antibodies) were used at a dilution of 1:2000. Bound antibodies were visualized by chemiluminescence using the ImmunoStar LD (290-69904) (Wako), and luminescent images were analyzed by a LuminoImager (LAS-3000; Fujifilm, Inc.). Only gels for CD63 (BD Biosciences) detection were run under non-reducing conditions.

##### Plasmids

psiRNA-LucGL3 was purchased from InvivoGen. Knockdown shRNA vector for human and mouse nSMase2 were purchased from TaKaRa Bio. A full-length human nSMase2 cDNA was cloned into pIRES2-EGFP vector (Clontech). Primary miR-210 were PCR-amplified from human genomic DNA and cloned into the downstream of CMV promoter in pIREShyg3 (Takara Bio). The sensor vector for miR-210 was constructed by introducing tandem binding sites with perfect complementarity to miR-210, separated by a four-nucleotide spacer into the XhoI site of psiCHECK2 (Promega). The sequences of the binding site are as follows: 5′-TTCTCGAGTTTCAGCCGCTGTCACACGCACAGTTACGCGTTTTCAGCCGCTGTCACACGCACAGTTCTCGAGTT-3′ (sense) and 5′-AACTCGAGAACTGTGCGTGTGACAGCGGCTGAAAACGCGTAACTGTGCGTGTGACAGCGGCTGAAACTCGAGAA-3′ (antisense). The “seed” sequence of miR-210 is *underlined*. In a mutated miR-210 sensor vector, the seed sequence, ACACGCA, was displaced with TGTGCGT. All of the plasmids were verified by DNA sequencing.

##### Isolation of RNAs

Isolation of exosomal and cellular RNAs was performed using the miRNeasy Mini Kit (Qiagen). Exosome or cell lysate was diluted with 1 ml of Qiazol solution. Subsequent extraction and filter cartridge work were carried out according to the manufacturer's protocol.

##### mRNA and miRNA Expression Analysis

The method for qRT-PCR has been described previously (10). PCR was carried out in 96-well plates using the 7300 Real Time PCR system (Applied Biosystems). All reactions were done in triplicate. All TaqMan MicroRNA assays were purchased from Applied Biosystems. RNU6 was used as an invariant control for the cells. Gene expression was analyzed using Taqman gene expression assays except primary miR-210 (Applied Biosystems). The expression levels of primary miR-210 and β-actin were measured by qRT-PCR using a SYBR Green PCR Master Mix (Invitrogen). Primer sequences are as follows (shown 5′ to 3′): primary miRNA-210, GACTGGCCTTTGGAAGCTCC (forward) and ACAGCCTTTCTCAGGTGCAG (reverse); β-actin, GGCACCACCATGTACCCTG (forward) and CACGGAGTACTTGCGCTCAG (reverse).

##### Nanoparticle Tracking Analysis

Nanoparticle tracking analysis was carried out using the Nanosight LM10-HS system (NanoSight) on exosomes resuspended in PBS and were further diluted for analysis. The results are presented as the average ± S.E. of three independent experiments.

##### Phase Contrast Electron Microscopy

A drop of the sample was put on a copper grid and coated with a carbon film with holes in it. Most of the liquid was removed with blotting paper, leaving a thin film stretched over the holes. The specimen was instantly shock-frozen by plunging into liquid ethane, which was cooled to 90 K by liquid nitrogen into a temperature-controlled freezing unit (Zeiss, Oberkochen, Germany). The remaining ethane was removed with blotting paper, and the specimen was transferred to the electron microscope. The phase plate was prepared from amorphous carbon films. The films were deposited by vacuum evaporation (JEOL JEE-400) on a freshly cleaved mica surface. For observation at 300-kV acceleration voltage, the film thickness corresponding to the *p*/two-phase plate was approximately 32 nm. At that thickness, the transparency of 300-kV acceleration electrons was estimated to be 70%. After preparation, the films were floated on the water's surface and then transferred to a molybdenum aperture with several holes 50-lm in diameter, which resulted in a cut-off frequency for special resolution of 0.5 nm. A hole approximately 0.5 lm in diameter in the center of the carbon film was used by a focused ion beam machine (JEOL JFIB-2000).

##### PKH67-labeled Exosome Transfer

Purified exosomes derived from 4T1 conditioned medium were labeled with a PKH67 green fluorescent labeling kit (Sigma-Aldrich). Exosomes were incubated with 2 μm PKH67 for 5 min, washed four times using 100-kDa filter (Microcon YM-100, Millipore) to remove excess dye, and incubated with HUVECs at 37 °C.

##### Microarray Analysis

To detect the miRNAs in exosomes and cells derived from HEK293, MCF10A, MCF7, and MDA-MB-231, 100 ng of total RNA was labeled and hybridized using a human microRNA microarray kit (Agilent Technologies) according to the manufacturer's protocol (protocol for use with Agilent MicroRNA microarrays, version 1.5). Hybridization signals were detected using a DNA microarray scanner (Agilent Technologies), and the scanned images were analyzed using Agilent Feature Extraction software.

##### Mouse Studies

Animal experiments in this study were performed in compliance with the guidelines of the Institute for Laboratory Animal Research, National Cancer Center Research Institute. Five- to seven-week-old female Balb/c athymic nude mice (CLEA Japan, Shizuoka, Japan) or SCID Hairless Outbred mice (Charles River Laboratories, Kanagawa, Japan) were anesthetized by exposure to 3% isoflurane for injections and *in vivo* imaging. We injected 4T1- or MDA-MB-231-D3H2LN- nSMase2-modified cells bilaterally into the subcutaneous (2 × 10^6^ cells were injected in 100-μl volume PBS) or mammary fat pad (2 × 10^6^ cells were injected in 50-μl volume Matrigel diluted with PBS) of anesthetized mice. We monitored mammary tumor growth by regular measurements using a digital caliper. After 3 to 4 weeks, we killed mice and determined metastasis in lungs by *ex vivo* or *in vivo* imaging. We carried out lung colonization assays by injecting 1 × 10^6^ 4T1-control or 4T1-nSMase2-KD cells (suspended in 100 μl of PBS) into the lateral tail vein. Lung colonization was studied and determined by *in vivo* luminescence imaging. For rescue experiment, 4T1-nSMase2-KD cells (2 × 10^6^ cells suspended in 100 μl of PBS) were subcutaneously injected. After 4 days of implantation, 1 μg of exosome was injected intratumoraly (100 μl in PBS) every other day for up to 18 days. Metastasis occurrence was determined by *in vivo* luminescence. For *in vivo* imaging, the mice were administered d-luciferin (150 mg/kg, Promega) by intraperitoneal injection. Ten minutes later, photons from animal whole bodies were counted using the IVIS imaging system (Xenogen) according to the manufacturer's instructions. Data were analyzed using LIVINGIMAGE software (version 2.50, Xenogen).

##### Statistics

Statistical analyses were performed using the Student's *t* test.

## RESULTS

### 

#### 

##### nSMase2 Regulates Cancer Cell Metastasis

**FIGURE 1.**
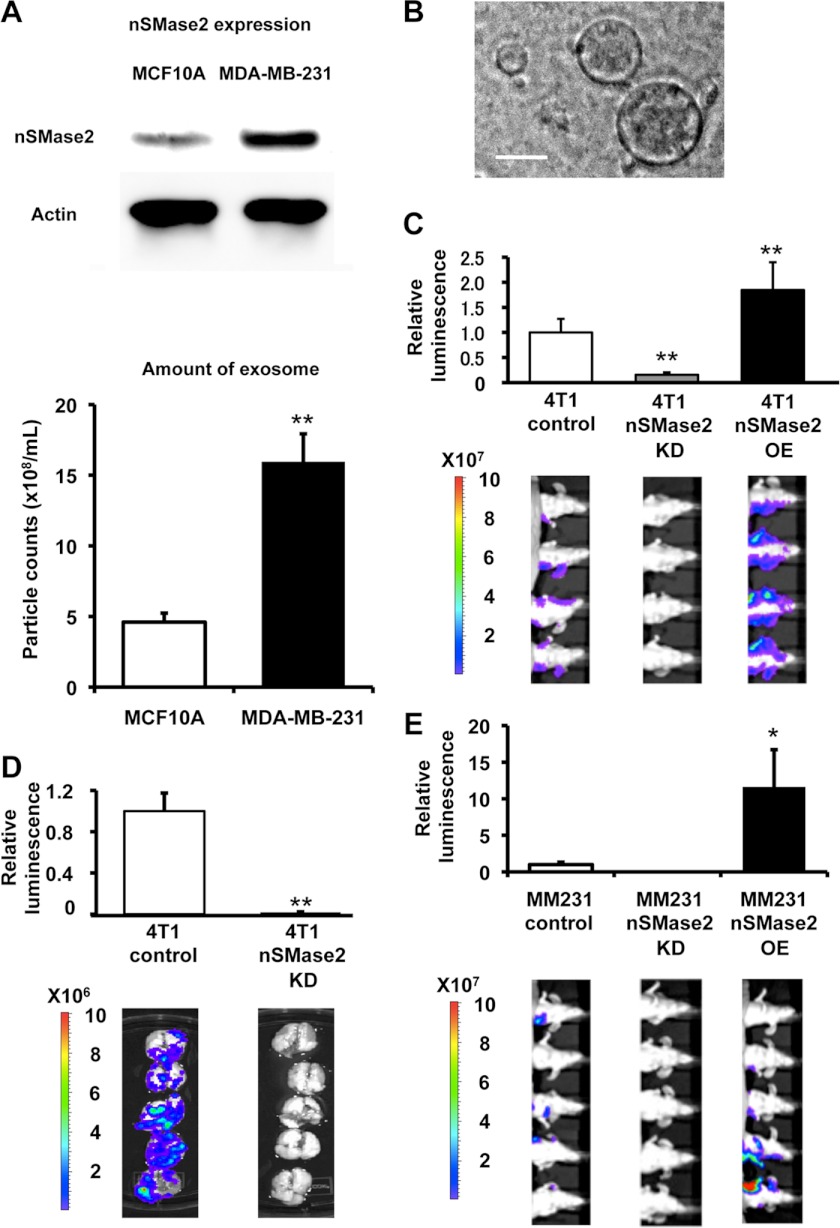
**nSMase2 regulates cancer cell metastasis.** *A*, the expression level of nSMase2 protein in (*upper panel*) and secretion level of exosome from (*lower panel*) MCF10A and MDA-MB-231 cells. The same number of cells was seeded. *Error bars* are presented as the mean S.E. (*n* = 3). **, *p* < 0.005, as compared with MCF10A cells. *B*, phase-contrast electron microscopy was used to image resuspend exosome pellets. *Scale bar*, 100 nm. *C*, bioluminescence quantification of lung metastasis by 4T1-control cells, 4T1-nSMase2-KD cells or 4T1-nSMase2-OE cells. Each *error bar* is presented as the mean S.E. (*n* = 4). **, *p* < 0.005, as compared with 4T1-control cells. *D*, luciferase activity in the lung, which was used to represent lung metastasis, was recorded for each mouse. Lung images from different mice are shown. Each *error bar* is presented as the mean S.E. (*n* = 5). **, *p* < 0.005, as compared with 4T1-control cells. *E*, bioluminescence quantification of metastasis by parental MDA-MB-231-D3H2LN (*MM231-control*) cells, MDA-MB-231-D3H2LN-nSMase2-OE (*MM231-nSMase2-OE*) cells, or MDA-MB-231-D3H2LN-nSMase2-KD (*MM231-nSMase2-KD*) cells. Each *error bar* is presented as the mean ± S.E. (*n* = 5). *, *p* < 0.05, as compared with MM231-control cells.

In a previous study, we have described how miRNAs are released through ceramide-dependent secretory machinery via the exosome (10). Specifically, we demonstrated that blocking the activity of nSMase2 resulted in reduced miRNA secretion and that nSMase2 overexpression led to increased levels of extracellular miRNAs (10, 11). In addition, we found that the expression level of nSMase2 was higher in cancer cells than that in non-cancer cells (Fig. 1*A*, *upper panel* and supplemental Fig. 1*A*). Furthermore, secretion level of exosome show correlation with the expression level of nSMase2 (Fig. 1*A*, *lower panel*, and supplemental Fig. 1*B*), suggesting that malignant cancer cells secrete more exosomes than non-cancer cells through the regulation of nSMase2. We confirmed that breast cancer cells secreted around 100-nm size of vesicles with a consistent size and uniform expression of known exosome marker, CD63 and HP70 (supplemental Fig. 1*C*) (18). Purified exosomes has also been shown by phase contrast electron microscopy and found that their size observed to be 90 ± 11.7 nm in diameter (*n* = 13) (Fig. 1*B*). To determine the role of nSMase2 in cancer cell malignancy, we employed 4T1 cells, which are mouse mammary tumor cells with a high tumorigenic and metastatic ability. Both stable nSMase2-knockdown and nSMase2-overexpressing 4T1 cells were generated (supplemental Figs. 1, *D* and *E*) and inoculated into mammary fat pad of the mice, and the tumors were subsequently evaluated for their metastatic colonization capacity in lung tissue. The expression of secretory miR-16 (supplemental Fig. 2*A*), which is known to abundantly existed in exosome, as well as exosome quantity, as determined by immunoblotting for exosome markers, HSP70 and Alix (supplemental Fig. 2*B*), protein concentration (supplemental Fig. 2*C*), and nanoparticle tracking analysis (supplemental Fig. 2*D*), decreased in nSMase2-knockdown cancer cells (4T1-nSMase2-KD cells) but increased in nSMase2-overexpressing cancer cells (4T1-nSMase2-OE cells). However, the expression of intracellular miRNAs was not altered in either of these established cell types (supplemental Figs. 2*A* and 3). After the orthotopic inoculation of these cell lines into mammary fat pad, we found that nSMase2 silencing in parental 4T1 breast cancer cells significantly decreased lung metastatic colonization (Fig. 1*C*), and *in vivo* imaging and histological observation revealed a significant decrease in the total number of metastatic nodules in nSMase2-knockdown lung tumors (Fig. 1*D* and supplemental Fig. 4*A*). In contrast, the overexpression of nSMase2 in 4T1 cells enhanced the metastatic capacity of these tumors (Fig. 1*C*). We also confirmed similar results using an orthotopic model of MDA-MB-231-D3H2LN cells, which are human breast cancer cells with a high metastatic ability, overexpressing or inhibiting nSMase2 (Fig. 1*E*), which suggests that the alteration in expression level of nSMase2 leads to the change in metastatic ability of cancer cells. Interestingly, nSMase2 inhibition or overexpression in 4T1 cells did not significantly enhance or inhibit cellular proliferation, invasion, or migration *in vitro* (supplemental Fig. 4*B*) and did not increase the mammary tumor volume (supplemental Fig. 4, *C* and *D*). In addition, no significant differences were found in expression profiles of cellular or miRNAs isolated from these nSMase2-modified cell lines (supplemental Fig. 3). Moreover, no significant reduction in metastatic potential was observed in the lungs of animals intravenously injected with parental 4T1 cells or 4T1-nSMase2-KD cells, which excludes the possibility that nSMase2 disruption affected the recruitment capacity of cancer cells to metastatic tissues (supplemental Fig. 5). These results indicate that the effect of nSMase2 on metastasis was not simply due to its effect on the cancer cells themselves.

##### Endothelial Activation Regulated by nSMase2-mediated Exosome Promotes Cancer Cell Metastasis

**FIGURE 2.**
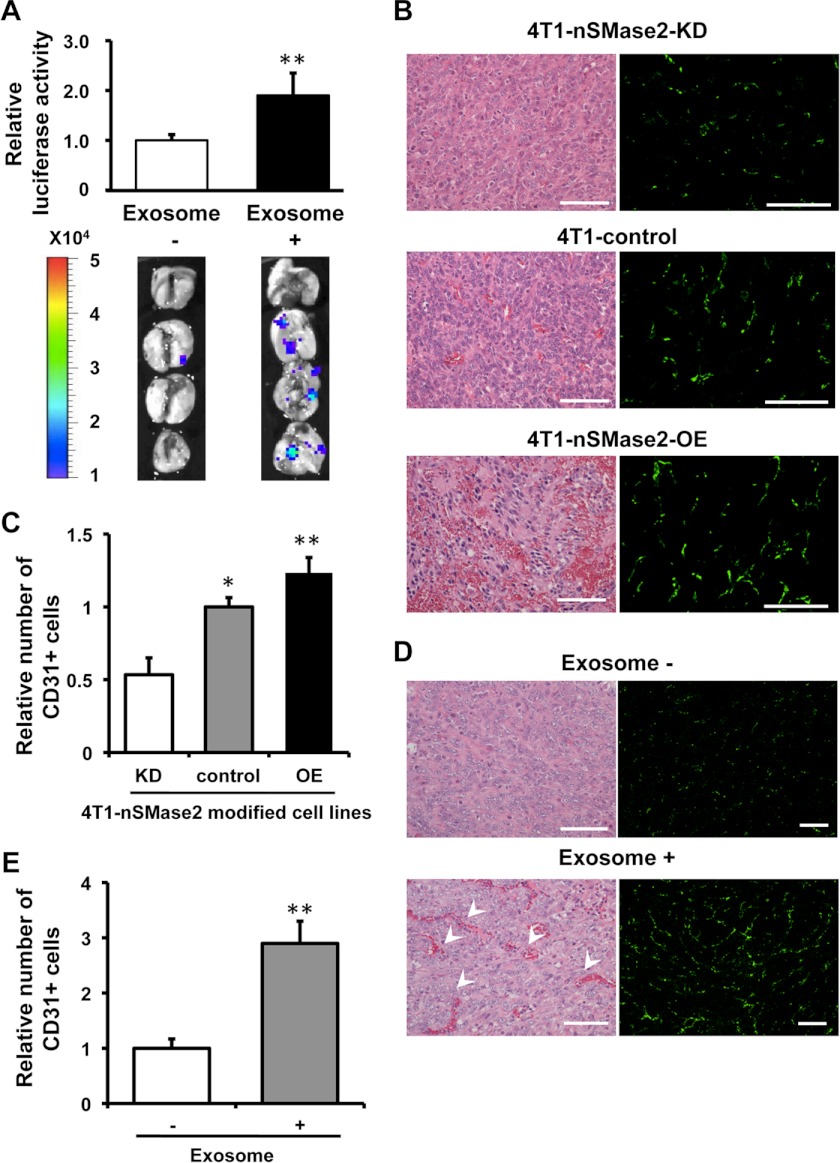
**Endothelial activation mediated by nSMase2 regulates cancer cell metastasis.** *A*, bioluminescence imaging of lung metastasis by 4T1-nSMase2-KD cells with or without the injection of exosomes isolated from parental 4T1 cells. Each *error bar* is presented as the mean ± S.E. (*n* = 4). **, *p* < 0.005, as compared with control injection. *B*, H&E of primary tumors isolated from parental 4T1-control cells, 4T1-nSMase2-KD cells or 4T1-nSMase2-OE cells (*left panel*). *Scale bars*, 100 μm for H&E. The endothelial cells were also evaluated using CD31 staining to detect blood vessels in tumors composed of parental 4T1 cells, 4T1-nSMase2-KD cells or 4T1-nSMase2-OE cells (*right panel*); *scale bars*, 100 μm. *C*, angiogenesis determined using CD31 staining which shown in *B* to detect blood vessels in tumors composed of parental 4T1 cells, 4T1-nSMase2-KD cells, or 4T1-nSMase2-OE cells, as above; *n* = 4 for each group. Each *error bar* is presented as the mean ± S.E. (*n* = 4). *, *p* < 0.05; **, *p* < 0.005, as compared with 4T1 control. *D*, H&E staining of primary tumors isolated from mice that received PBS or an injection of exosomes from parental 4T1 cells following the transplantation of 4T1-nSMase2-KD cells (*left panel*). *Arrowheads* show red blood cells in vascular structure. *Scale bars*, 100 μm for H&E. The endothelial cells were also evaluated using CD31 staining to detect blood vessels in tumors composed of 4T1-nSMase2-KD cells with or without exosome injection (*right panel*); *scale bars*, 200 μm. *E*, angiogenesis determined using CD31 staining, which was shown in *D* to detect blood vessels in tumors composed of 4T1-nSMase2-KD cells with or without exosome, as above; *n* = 4 for each group. Each *error bar* is presented as the mean ± S.E. (*n* = 4). **, *p* < 0.005, as compared with control injection.

Consistent with a role for nSMase2 in the initiation of metastasis, intratumor injection of exosomes isolated from parental 4T1 cells to non-metastatic 4T1-nSMase2-KD cells after orthotopical inoculation into mammary fat pad significantly enhanced their metastatic colonization (Fig. 2*A* and supplemental Fig. 6*A*), whereas the growth of the inoculated 4T1-nSMase2-KD tumor cells was unaffected (supplemental Fig. 6*B*). These results indicated that endogenous nSMase2 could act to enhance metastatic initiation through the secretion of exosomes. When examining the selective disadvantage provided by nSMase2 silencing in cancer cells, we noticed that blood vessels were difficult to detect in animals that received 4T1-nSMase2-KD cells (Fig. 2*B*, *left panel*). As a result, we hypothesized that tumors inoculated with nSMase2-knockdown cells would display reduced blood vessel densities upon microscopic visualization of the primary tumor after staining for the endothelial marker CD31. The imaging analysis revealed that primary tumors derived from 4T1-nSMase2-KD cells had significantly lower endothelial cell densities than did tumors derived from control cells (Fig. 2*B*, *right panel*, and 2*C*). In contrast, tumors derived from 4T1-nSMase2-OE cells displayed higher endothelial densities than did tumors derived from control cells (Fig. 2*B*, *right panel*, and 2*C*). In addition, there were increased numbers of endothelial cells in tumors derived from 4T1-nSMase2-KD cells that were subsequently injected with parental 4T1 cell-derived exosomes compared with control treatment (Fig. 2, *D* and *E*). Thus, these observations indicate that release of nSMase2-mediated exosome enhances endothelial cell density, whereas the inhibition of nSMase2 provides metastatic cells with a selective disadvantage for endothelial interactions and angiogenic progression.

##### Exosomes Derived from Metastatic Cancer Cells Enhances Activity of Endothelial Cells

**FIGURE 3.**
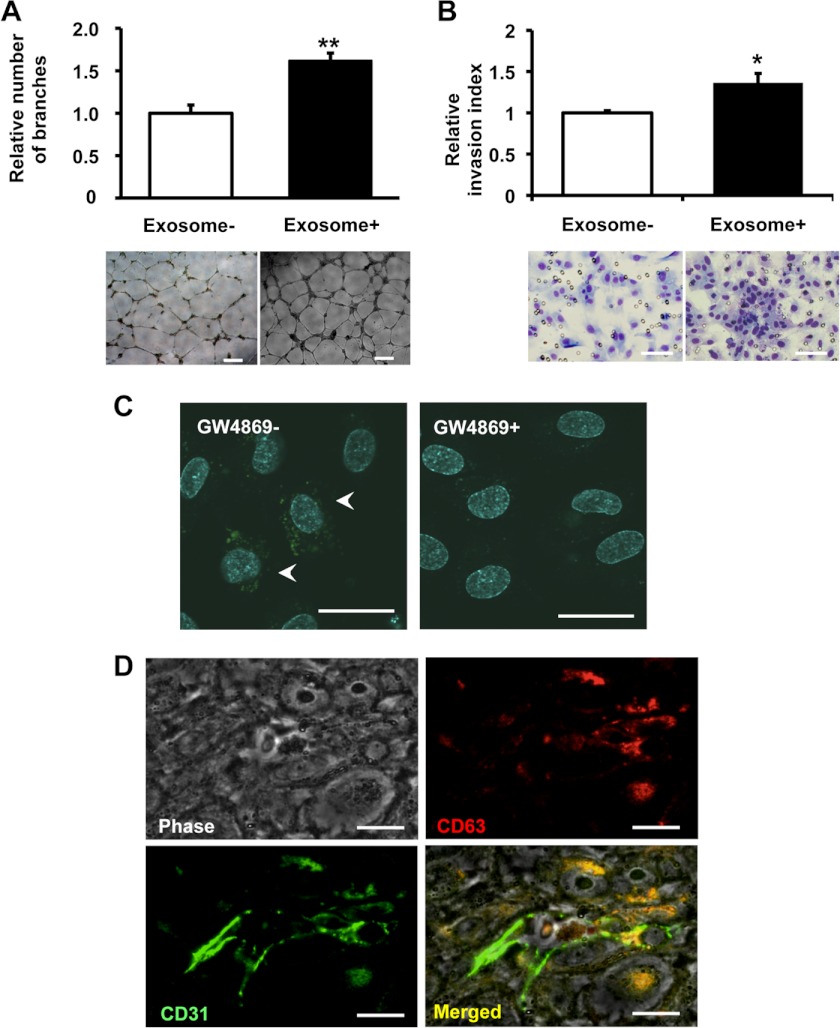
**Exosomes derived from metastatic cancer cells enhances activity of endothelial cells.** *A*, capillary tube formation in endothelial cells seeded onto Matrigel following the addition of exosomes from parental 4T1 cells. A representative image at 16 h after plating is shown, including the quantification of the average number of branches at 16 h after plating. The *scale bar* indicates 500 μm. *B*, the effect of exosome on HUVEC migration was determined by Transwell migration assay. A representative image at 48 h after plating is shown, including the quantification of the average number of migrated HUVECs at 48 h after plating. The *scale bar* indicates 100 μm. *C*, an *in vitro* co-culture system was used, whereby 4T1 cells were seeded in the *top compartment* and separated from HUVECs in the *bottom compartment* by a porous membrane. 4T1 cells (*top compartment*) were transduced with a CD63-GFP vector and co-cultured with HUVECs (*bottom compartment*). *Scale bars*, 100 μm. *D*, immunostaining of CD31 (*green*) and CD63 (*red*) on 4T1-hCD63 inoculated tumor. The *scale bar* indicates 10 μm. CD63 is co-localized with CD31-positive endothelial cells.

We next sought to determine the cellular basis for nSMase2-regulated exosome-dependent angiogenesis. For this purpose, we first evaluated the effect of exosome from parental 4T1 cells in HUVECs. As a result, although cellular proliferation of HUVECs was slightly increased by the addition of 4T1 exosome (supplemental Fig. 7*A*), addition of purified exosomes derived from metastatic 4T1 cells enhanced not only tube formation in HUVECs, as assessed by the quantification of branch points (Fig. 3*A*), but also migration of HUVECs (Fig. 3*B*). Next, to determine whether exosomes secreted by metastatic breast cancer cells could be incorporated in a paracrine manner, we employed a co-culture system for HUVECs and 4T1 cells, in which the cells are separated by a membrane with a 0.4-μm pore size to prevent direct cell contact or the transfer of larger vesicles. In this experiment, we used 4T1 cells that had been transduced with a CD63-GFP fusion gene, and we analyzed GFP fluorescence present in HUVECs after 3 days of co-culture by confocal microscopy. These studies showed that exosomes could be transferred from breast cancer cells to endothelial cells during co-culture (Fig. 3*C*, *left panel*). However, the transfer of exosomes from cancer cells to endothelial cells was completely abolished by the addition of nSMase2 inhibitor, GW4869 that was reported to inhibit the secretion of exosome from cells (10, 19), to the 4T1-CD63-GFP cells (Fig. 3*C*: right panel). In addition, 4T1 exosomes labeled with the fluorescent dye PKH67 were cultured with HUVECs and were found to be internalized into endosome-like structures by endothelial cells (supplemental Fig. 7*B*). To confirm whether the exosomes from inoculated cancer cells were incorporated into endothelial cells *in vivo*, an immunohistochemical analysis was performed following the inoculation of 4T1-hCD63 cells *in vivo* (Fig. 3*D*). As shown in Fig. 3*D*, the CD31-positive cells (*green*) was co-localized with CD63 (*red*) signals in the tumor. These findings reveal that enhanced tube formation in endothelial cells is a key feature of metastatic breast cancer cell populations that is regulated in a humoral fashion by exosomes released from metastatic cancer cells.

##### Exosomal Angiogenic miRNAs from Cancer Cells Regulate Angiogenesis in Endothelial Cells

**FIGURE 4.**
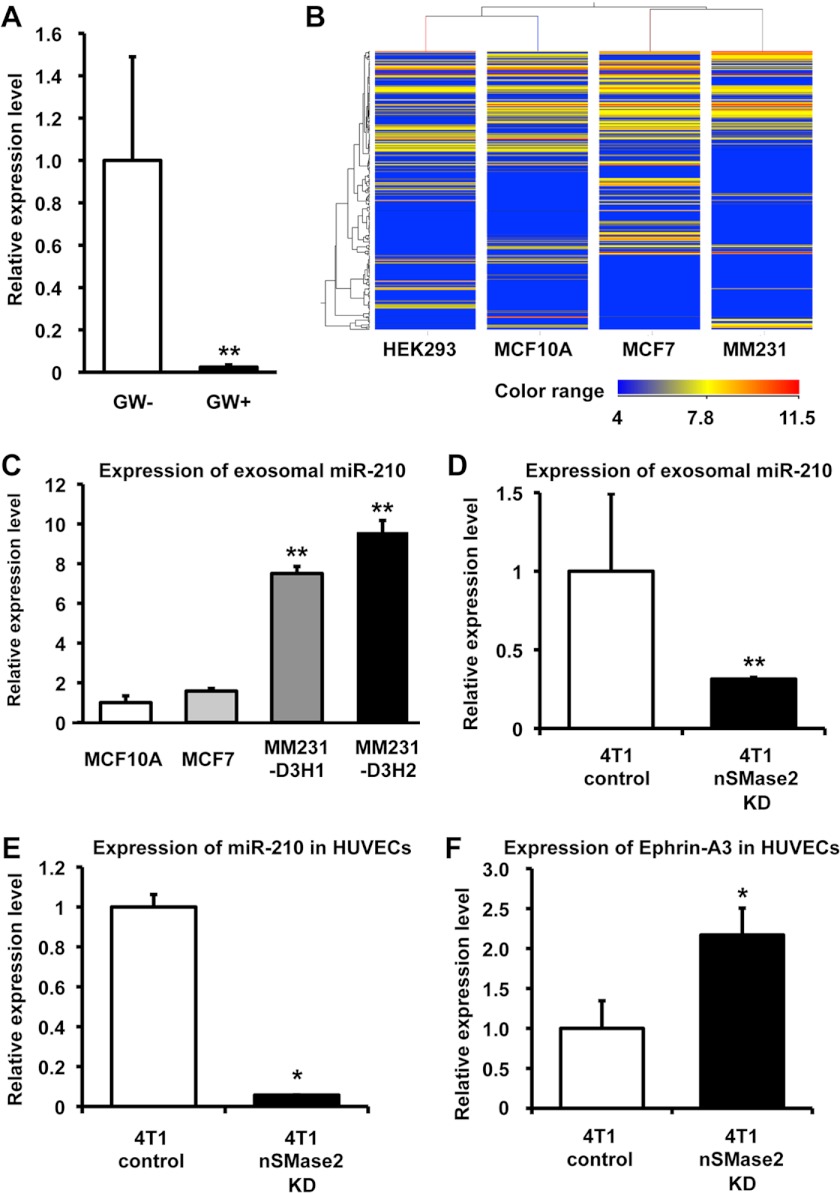
**Exosomal angiogenic miRNAs from cancer cells regulate angiogenesis in endothelial cells.** *A*, 4T1-siLuc cells were treated with 10 μm GW4869 at the start of the co-culture for a total of 48 h (*p* < 0.001). Each *error bar* is presented as the mean ± S.E. (*n* = 3). **, *p* < 0.005, as compared with control. *B*, heat map showing expression levels of the exosomal miRNAs isolated from HEK293, MCF10A, MCF7, and MDA-MB-231. *Blue* to *red*, color range gradient of mean abundance. *C*, the expression level of miR-210 in exosome isolated from MCF10A, MCF7, MDA-MB-231-D3H1 (MM231-D3H1), or MDA-MB-231-D3H2LN (MM231-D3H2) cells. Each *error bar* is presented as the mean ± S.E. (*n* = 3). **, *p* < 0.005, as compared with MCF10A. *D*, expression of exosomal miR-210 in exosomes isolated from parental 4T1 cells or 4T1-nSMase2-KD cells. Each *error bar* is presented as the mean ± S.E. (*n* = 4). **, *p* < 0.005, as compared with 4T1-control cells. *E*, HUVECs were co-cultured with parental 4T1 cells or 4T1-nSMase2-KD cells for 48 h. RNA was isolated from the HUVECs at 48 h after the start of co-culture, and the expression of exosomal miR-210 in the HUVECs was analyzed by qRT-PCR. Each *error bar* is presented as the mean ± S.E. (*n* = 3). *, *p* < 0.05, as compared with 4T1 control cells. *F*, parental 4T1 cells or 4T1-nSMase2-KD cells were co-cultured with HUVECs for 48 h, and the expression levels of ephrin-A3 (target of miR-210) were analyzed by qRT-PCR. Each *error bar* is presented as the mean ± S.E. (*n* = 3). *, *p* < 0.05, as compared with 4T1-control cells.

It is well known that angiogenic miRNAs regulate multiple endothelial cell functions and that nSMase2 is essential for miRNA secretion from cells (10, 20, 21). These reports, in addition to our findings described above, prompted us to evaluate the hypothesis that exosomal miRNAs from cancer cells are responsible for this phenomenon. To prove this hypothesis, we used 4T1 cells that had been transduced with a luciferase short hairpin RNA-overexpressing vector (4T1-siLuc). This established cell line secretes luciferase siRNA molecules with a nucleic acid sequence not present in the mammalian genome (supplemental Fig. 8*A*). To evaluate whether the transfer of luciferase siRNA occurred in the form of exosome transfer, we added GW4869 to co-cultured 4T1-siLuc cells and assessed the transfer of luciferase siRNA to HUVECs by qRT-PCR. Although we were able to measure luciferase siRNA in control-treated HUVECs, this siRNA sequence was minimally detected in HUVECs co-cultured with GW4869 treated 4T1-siLuc cells (Fig. 4*A*), which indicates that small RNAs, including not only siRNA but also miRNA, could in fact be transferred from cancer cells to endothelial cells during co-culture and that the transfer of small RNAs is mediated by exosome release regulated by nSMase2. We next sought to determine whether exosomal miRNAs could selectively regulate the angiogenesis of endothelial cells. To address this hypothesis, we performed a miRNA microarray analysis of the following four cell lines: metastatic breast cancer cells (MDA-MB-231); non-metastatic cancer cells (MCF7); normal mammary epithelial cells (MCF10A); and human embryonic kidney cells (HEK293). The microarray analysis of miRNA populations in exosomes isolated from these cell lines were performed using a miRNA microarray (Fig. 4*B*). Interestingly, some of the exosomal miRNAs that were highly enriched in the metastatic cancer cell line are known to regulate angiogenesis in endothelial cells (22). One of these miRNAs, miR-210, which is well known as an angiogenic miRNA, and its expression was correlated with poor prognosis in breast cancer (23, 24). Moreover, a recent report showed that high expression levels of miR-210 in plasma are associated with the presence of tumor in patients with breast cancer and with trastuzumab resistance in patients with HER2-positive breast cancer (25). In addition, the expression level of miR-210 was significantly higher in breast cancer patients with lymph node metastasis than in breast cancer patients without lymph node metastasis (25). From our data and previous reports, because the contribution of exosome against cancer metastasis was mediator of endothelial activation, we postulated that exosomal miR-210 might be one of the regulators in exosome for the angiogenesis around the cancer cells. Indeed, miR-210 expression in exosome was higher in malignant cancer cells than that in non-malignant cancer cell or non-cancer cells (Fig. 4*C*). To confirm that exosomal miR-210 were down-regulated in nSMase2-impaired cancer cells, we performed a qRT-PCR analysis for these cells. As shown in Fig. 4*D*, the expression of exosomal miR-210 was down-regulated in nSMase2 knockdown cells when compared with control cells, although the cellular levels of the miRNAs were not altered (supplemental Fig. 8*B*). Moreover, we performed a co-culture experiment using 4T1-nSMase-KD cells or parental 4T1 cells with HUVECs and then measured the expression of miR-210 in the HUVECs. Co-culture with parental 4T1 cells, compared with 4T1-nSMase-KD cells, led to the higher detection of miR-210 in HUVECs (Fig. 4*E*), indicating that exosomal miR-210 from metastatic cancer cells transfer to recipient endothelial cells. Then, we employed this co-culture system to study the effects of exosomes isolated from parental 4T1 cells on the expression of the established miR-210 target gene, ephrin-A3 (26). The presence of parental 4T1 cells reduced the expression level of ephrin-A3 in HUVECs compared with 4T1-nSMase2-KD cells (Fig. 4*F*). To exclude the possibility that the exosome from cancer cells itself induces the endogenous expression of miR-210 in HUVECs, we quantified the expression of primary miR-210 in HUVECs co-cultured with parental 4T1 cells or 4T1-nSMase-KD cells. As shown in supplemental Fig. 8*C*, we did not find any difference of primary miR-210 expression level between HUVECs co-cultured with parental 4T1 cells or 4T1-nSMase-KD cells, although the expression of primary miR-210 levels was induced 20-fold above basal levels by desferrioxamine, which is an iron chelator and known to induce the expression of hypoxia inducible factor-1α (27), treatment compared with untreated cells (supplemental Fig. 8*D*). Taken together, these results suggest that the enhanced angiogenesis mediated by exosomes isolated from metastatic cancer cells is due to the presence of angiogenic miRNAs within the exosomes.

##### Exosomal miR-210 Enhanced Angiogenic Activity in Endothelial Cells in Vitro

**FIGURE 5.**
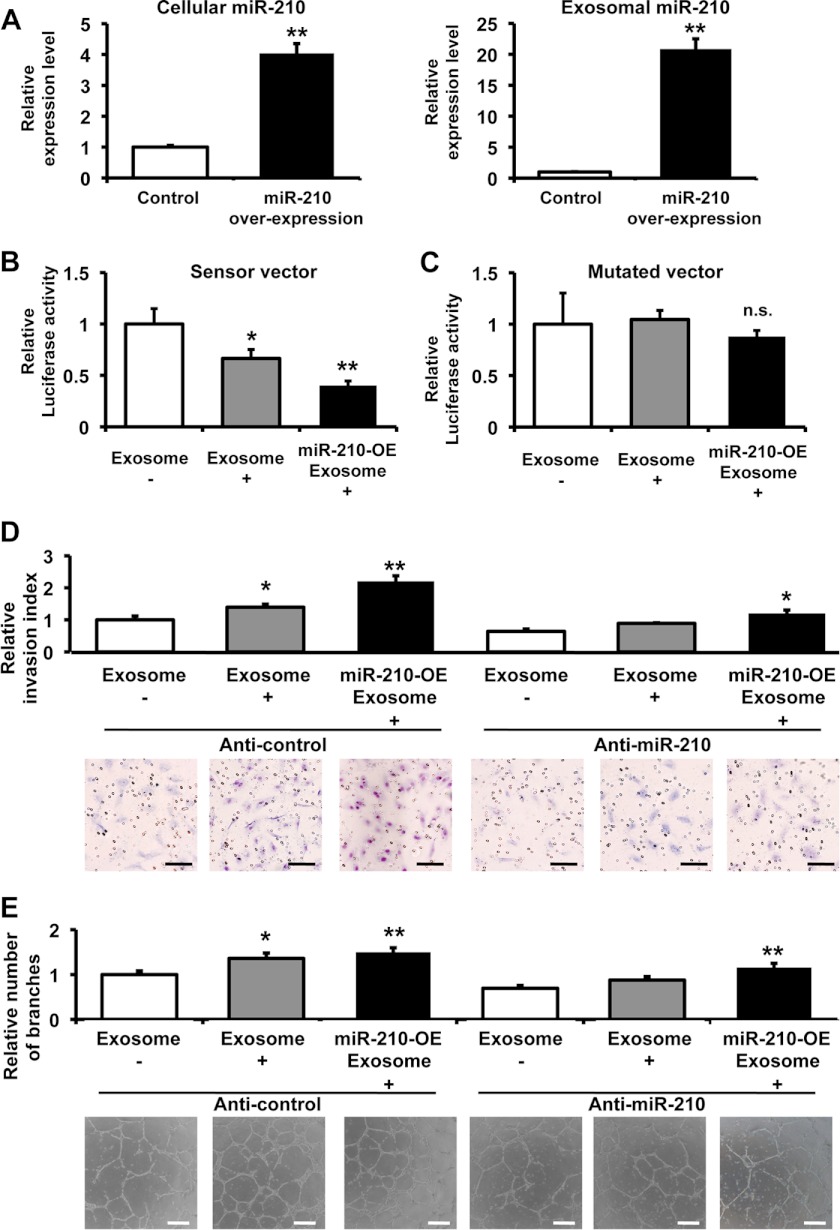
**Exosomal miR-210 from cancer cells enhanced the angiogenesis in endothelial cells.** *A*, the expression level of miR-210 in the cells (*left panel*) and exosome (*right panel*) from miR-210 overexpressing cells and control vector transfected cells. Each *error bar* is presented as the mean ± S.E. (*n* = 3). **, *p* < 0.005, as compared with control. *B*, Exosome derived from 4T1 cells suppressed the luciferase activity of the sensor vector. HUVECs transfected with an miR-210 sensor vector were used as recipient cells. The recipient cells were incubated in an miR-210-enriched exosome, control exosome, or PBS. After a 1-day incubation, a luciferase reporter assay was performed. The values on the *y* axis are depicted relative to the normalized luciferase activity of control PBS-treated cells, which is defined as 1. Each *error bar* is presented as the mean ± S.E. (*n* = 5). *, *p* < 0.05; **, *p* < 0.005, as compared with control. *C*, exosome did not reduce the luciferase activity of the mutated sensor vector. HUVECs transfected with the mutated miR-210 sensor vector were used as recipient cells. The recipient cells were incubated in an miR-210-enriched exosome, control exosome, or PBS. The luciferase assay was carried out as described above. The values on the *y* axis are depicted relative to the normalized *Renilla* luciferase activity of control cells, which is defined as 1. Each *error bar* is presented as the mean ± S.E. (*n* = 4). *n.s.* represents not significant. *D*, the transfection of anti-miR-210 to HUVECs inhibited the induction of capillary formation by exosomes derived from 4T1 cells. Following transfection with 3 nm of the miR-210 inhibitory molecule (anti-miR-210) or a control molecule (anti-NC), the HUVECs were incubated for 1 day, and these cells were then assessed using the migration assay with miR-210-enriched exosomes, control exosomes, or PBS. A representative image at 48 h after plating is shown, including the quantification of the average number of migrated HUVECs at 48 h after plating. Each *error bar* is presented as the mean ± S.E. (*n* = 3). *, *p* < 0.05; **, *p* < 0.005 as compared with PBS treatment. The *scale bar* indicates 100 μm. *E*, capillary tube formation in endothelial cells seeded onto Matrigel following the addition of miR-210-enriched exosomes, control exosomes, or PBS. A representative image at 16 h after plating is shown, including the quantification of the average number of branches at 16 h after plating. Each *error bar* is presented as the mean ± S.E. (*n* = 3). *, *p* < 0.05; **, *p* < 0.005 as compared with PBS treatment. The *error bar* indicates 500 μm.

To show the direct evidence that exosomal miR-210 released from cancer cells contributed to the enhancement of endothelial function in HUVECs, we collected miR-210 enriched exosome, which was isolated from miR-210 transiently transfected 4T1 cells. After the transfection of miR-210 expression vector to 4T1 cells, its expression was increased not only in the cells (Fig. 5*A*, *left panel*) but also in the exosomes (Fig. 5*A*, *right panel*). To confirm whether the transferred miR-210 are functional in the recipient HUVECs or not, we performed an miRNA-responsive reporter assay. We implemented luciferase analyses using a sensor vector harboring *Renilla* luciferase tandemly fused with miR-210 antisense sequence in the 3′-UTR. As shown in Fig. 5*B*, the normalized *Renilla* luciferase activities were reduced by the addition of exosome derived from 4T1 cells. Furthermore, miR-210-enriched exosome suppressed luciferase activity more effective than original exosome (Fig. 5*B*). In contrast, we did not detect any changes of luminescence by using a mutated vector instead of the intact sensor vector (Fig. 5*C*), indicating that exosomal miR-210 transferred and functional in recipient endothelial cells. Although the cellular proliferation of HUVECs was only slightly induced by the addition of miR-210-enriched exosome (supplemental Fig. 9), migration and capillary formation of HUVECs were significantly enhanced by the addition of miR-210-enriched exosome (Fig. 5, *D* and *E*). Though miR-210 inhibitory molecule (anti-miR-210) inhibited capillary formation and migration of HUVECs treated by exosome, and this inhibition was partially overcome by the addition of miR-210 enriched exosome (Fig. 5, *D* and *E*), indicating that miR-210 in exosome had a function to modulate endothelial activation. Taken together, these results illustrate that the transfer of exosomal miR-210 from metastatic cancer cells to endothelial cells is regulated by cancer cell nSMase2 expression and the activation of endothelial cells to overcome their niche for their benefit.

## DISCUSSION

Our data indicate that nSMase2 can activate exosomal miRNA secretion, which contributes to cancer cell metastasis through the induction of angiogenesis in the tumor microenvironment. These findings establish a key role for cancer cell-endothelial cell interactions for the initiation of metastasis.

Open questions remain regarding the physiological importance of exosome, however, the evidences for the contribution of exosome in cancer malignancy have been accumulating. For instance, exosomes from highly metastatic melanoma cells increased the metastatic behavior of primary tumors by educating bone marrow progenitors through the receptor tyrosine kinase MET (28). Although the number of exosomes did not differ based on clinical stage of melanoma patients, exosome protein concentrations were higher in subjects with stage 4 disease compared with other stages and to normal controls (28). Furthermore, exosome from metastatic breast cancer cells induced the mobilization of a population of neutrophil immune cells (29). Thus, all of these studies showed the possible involvement of angiogenic exosome to promote cancer metastasis. In the present study, we have found that exosomal angiogenic miRNAs, such as miR-210, regulate the metastatic ability of cancer cells. Considering that the circulating miR-210 level was increased in the serum of cancer patients with malignant breast cancer (25), exosomal angiogenic miR-210 might be one of the key factors for the tumor angiogenesis in the pathophysiological condition.

It has been known that nSMase2, which generates ceramide production in the cells, regulates multiple cellular activities in the cells via ceramide signaling. For instance, nSMase2 has been reported to act as a growth suppressor in MCF7 cells (30). On the contrary, nSMase2 was activated by Urokinase-type plasminogen activator triggering interaction of integrin α_v_β_3_, Urokinase-type plasminogen activator receptor, and matrix metalloproteinases, resulting in the induction of cellular proliferation (31). These reports suggest that the effects of nSMase2 up-regulation or down-regulation depend on the cellular origin and situation. In this article, we clearly showed that modulation of nSMase2 affect the exosome production from mouse mammary tumor cells lines 4T1 cells and human breast cancer cell lines, MDA-MB-231 cells. To further understand the exosome-mediated cancer progression, it is essential to examine whether nSMase2 regulate the exosome production in every types of cancer cells or not.

miRNAs were known to affect the expression of multiple target genes. For this reason, we could not rule out the possibility that miR-210 overexpression induced the angiogenic factors in exosome. In this work, we prepared the “miR-210-enriched exosome” by transient transfection and collected the exosome within 2 day after the transfection of miR-210 vector to try to avoid the effect of miR-210 in cancer cells. In addition, the effect of exosome was partially cancelled by the introduction of miR-210 inhibitor in HUVECs (Fig. 5, *D* and *E*). Furthermore, miR-210-enriched exosome overcome the inhibitory activity of miR-210 inhibitor in HUVECs (Fig. 5, *D* and *E*). These results suggest that the miR-210 in exosome from cancer cells can be incorporated in endothelial cells cells via exosomes, and this transferred miR-210 itself suppress their target genes, resulting in the activation of endothelial cells.

In conclusion, we propose that cancer cells provide nSMase2-regulated exosomal miRNAs to endothelial cells to promote their metastatic initiation efficiency. This work is the first to connect cancer metastasis to the nSMase2-mediated exosome *in vivo* and demonstrates that exosome-mediated metastasis occurs via the enhancement of microenvironmental angiogenesis by exosomal miRNAs. To understand the molecular mechanism of this on-demand system should also shed light on novel approaches for cancer therapy through the inhibition of angiogenesis.

## Supplementary Material

Supplemental Data

## Footnotes

3 The abbreviations used are:

miRNA: microRNA
nSMase2: neutral sphingomyelinase 2
KD: knockdown
luc: luciferase
HUVEC: human umbilical cord vein endothelial cell
nSMase2-OE: nSMase2-overexpressing cancer cells
qRT-PCR: quantitative RT-PCR.
