# Supplementary material for: Neutral Sphingomyelinase 2 (nSMase2)-dependent Exosomal Transfer of Angiogenic MicroRNAs Regulate Cancer Cell Metastasis
Source: J Biol Chem. 2013 Feb 25;288(15):10849–59. doi: 10.1074/jbc.M112.446831 (PMC3624465; doi:10.1074/jbc.M112.446831)
Supplement: Supplemental Data [file supp_288_15_10849__index.html]

Neutral sphingomyelinase 2 (nSMase2)-dependent exosomal transfer of angiogenic microRNAs regulate cancer cell metastasis — Neutral Sphingomyelinase 2 (nSMase2)-dependent Exosomal Transfer of Angiogenic MicroRNAs Regulate Cancer Cell Metastasis — Exosomal Angiogenic miRNAs from Cancer Cells — Supplemental Data 

# Neutral Sphingomyelinase 2 (nSMase2)-dependent Exosomal Transfer of Angiogenic MicroRNAs Regulate Cancer Cell Metastasis

## Supplemental Data

**Files in this Data Supplement:**

- Supplemental Figures (.pdf, 6.9 MB) - Supplemental figures including figure 1-9
