# Supplementary material for: Neutral Sphingomyelinase 2 (nSMase2)-dependent Exosomal Transfer of Angiogenic MicroRNAs Regulate Cancer Cell Metastasis
Source: J Biol Chem. 2013 Feb 25;288(15):10849–59. doi: 10.1074/jbc.M112.446831 (PMC3624465; doi:10.1074/jbc.M112.446831)
Supplement: Supplemental Data [file supp_M112.446831_jbc.M112.446831-1.pdf]

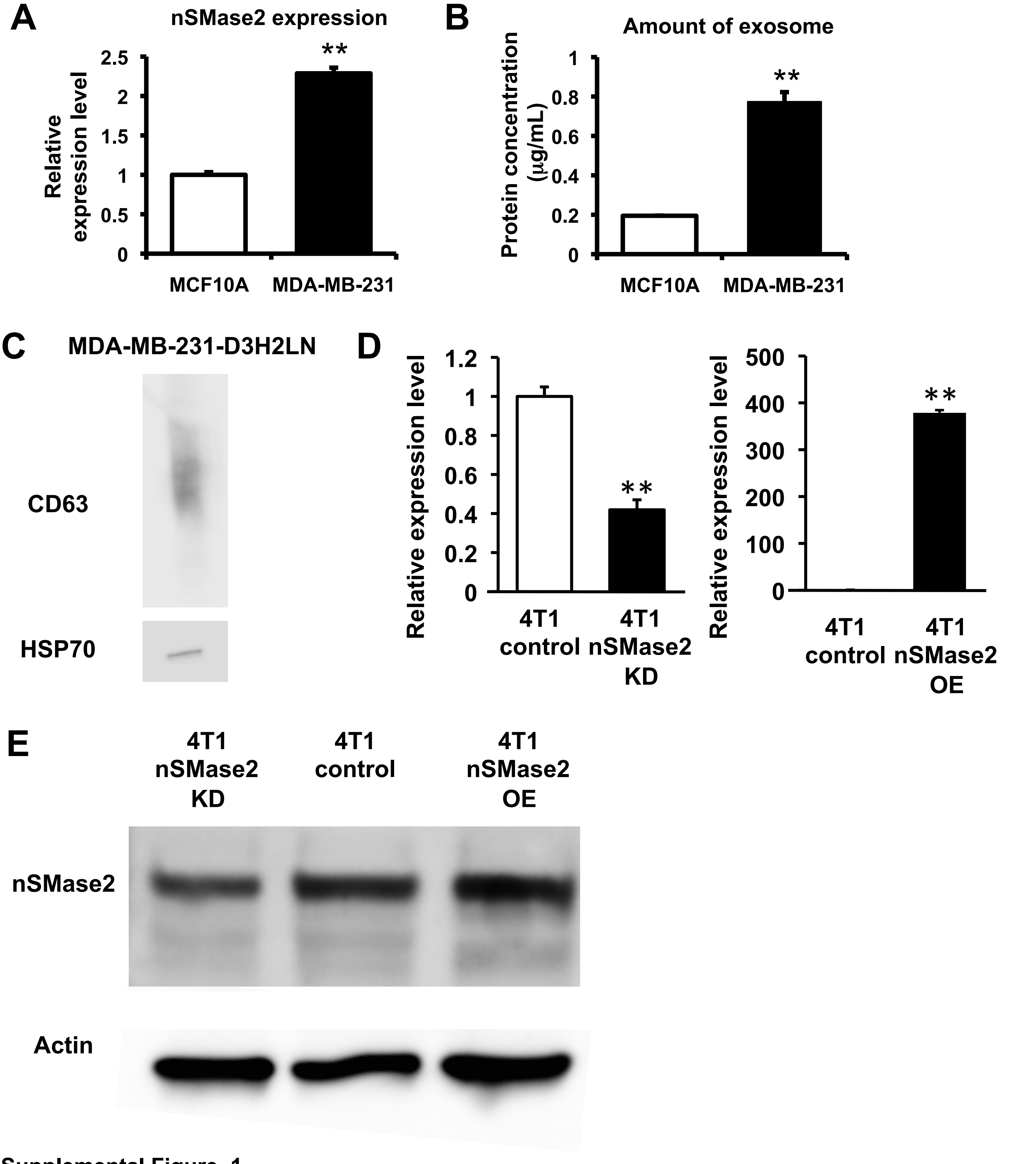

Supplemental Figure. 1

(A) The expression level of nSMase2 mRNA in MCF10A and MDA-MB-231 cells. Each bar is presented as the mean S.E. (n=3). **, p<0.005, as compared with MCF10A cells. (B) The secretion level of exosome from MCF10A and MDA-MB-231 cells. The same number of cells was seeded. Each bar is presented as the mean S.E. (n=3). **, p<0.005, as compared with MCF10A cells. (C) Immunoblotting of CD63 and HSP70 for exosomes derived from MDA-MB-231-D3H2LN. (D) The expression of nSMase2 in established 4T1-control cells, 4T1-nSMase2-KD cells (left panel) and 4T1-nSMase2-OE cells (right panel). Each bar is presented as the mean S.E. (n=3). **, p<0.005, as compared with 4T1-control cells. (E) Immunoblotting of nSMase2 and actin derived from parental 4T1-control, 4T1-nSMase2-KD cells and 4T1-nSMase2-OE cells. Equal volumes of protein concentration were analyzed by western blotting.

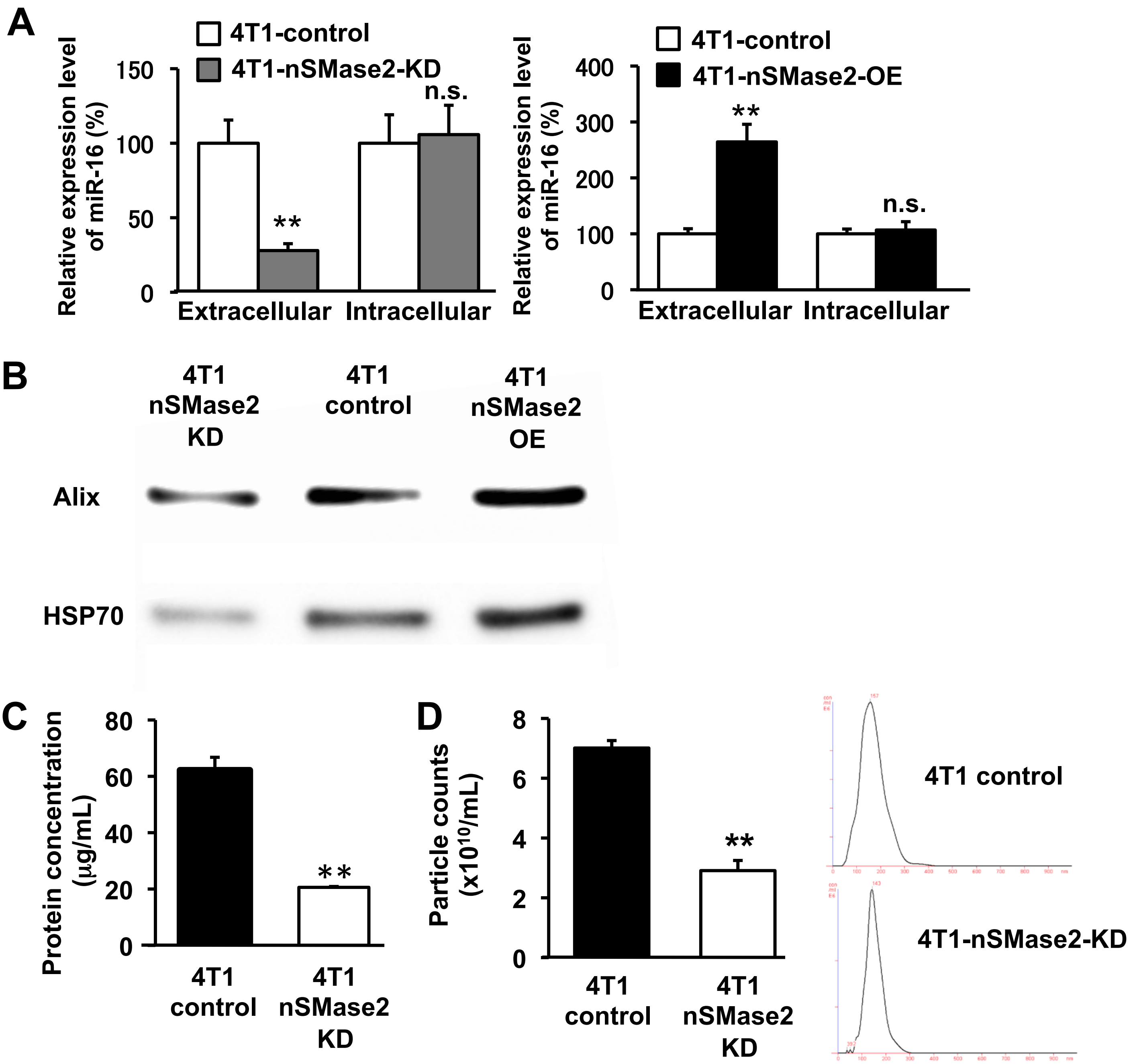

Supplemental Figure. 2

(A) Stable cell lines were established using nSMase2-shRNA or nSMase2-overexpression vectors. The left panel shows the secreted level of extracellular miRNAs and the intercellular level of miRNAs in 4T1-nSMase2-KD cells and parental 4T1-control cells. The right panel shows the secreted level of extracellular miRNAs and the intercellular level of miRNAs in 4T1-nSMase2-OE cells and parental 4T1-control cells. Each bar is presented as the mean S.E. (n=3). **, p<0.005, as compared with 4T1-control cells. *n.s.* represents not significant. (B) Immunoblotting of Alix and HSP70 for exosomes derived from parental 4T1-control, 4T1-nSMase2-KD cells and 4T1-nSMase2-OE cells. Equal volumes of exosomes were isolated from same numbers of cells and analyzed by western blotting. (C) The protein levels of exosomes derived from 4T1-control and 4T1-nSMase2-KD cells. Equal volumes of exosomes were isolated from same numbers of cells and analyzed by microBCA assay. Each bar is presented as the mean S.E. (n=3). **, p<0.005, as compared with 4T1-control cells. (D) The numbers of exosomes from 4T1-control and 4T1-nSMase2-KD cells were measured by nanoparticle tracking analysis. The size distribution of the exosomes from both cells were approximately 140 nm in diameter. Each bar is presented as the mean S.E. (n=3). **, p<0.005, as compared with 4T1-control cells.

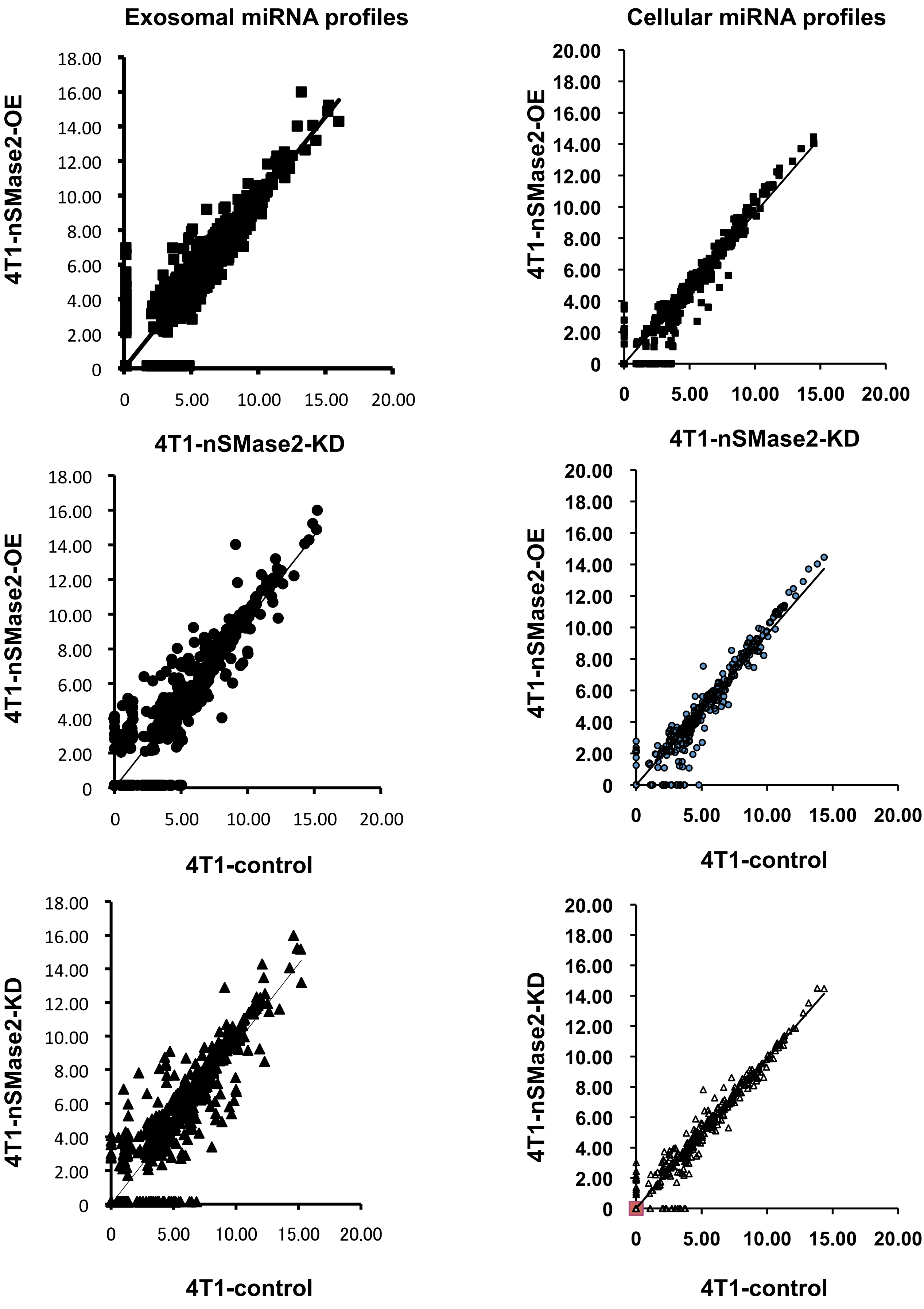

Supplemental Figure. 3
Linear scatter plot of gene expression from cellular and exosomal miRNAs among parental 4T1-control, 4T1-nSMase2-KD cells and 4T1-nSMase2-OE cells.

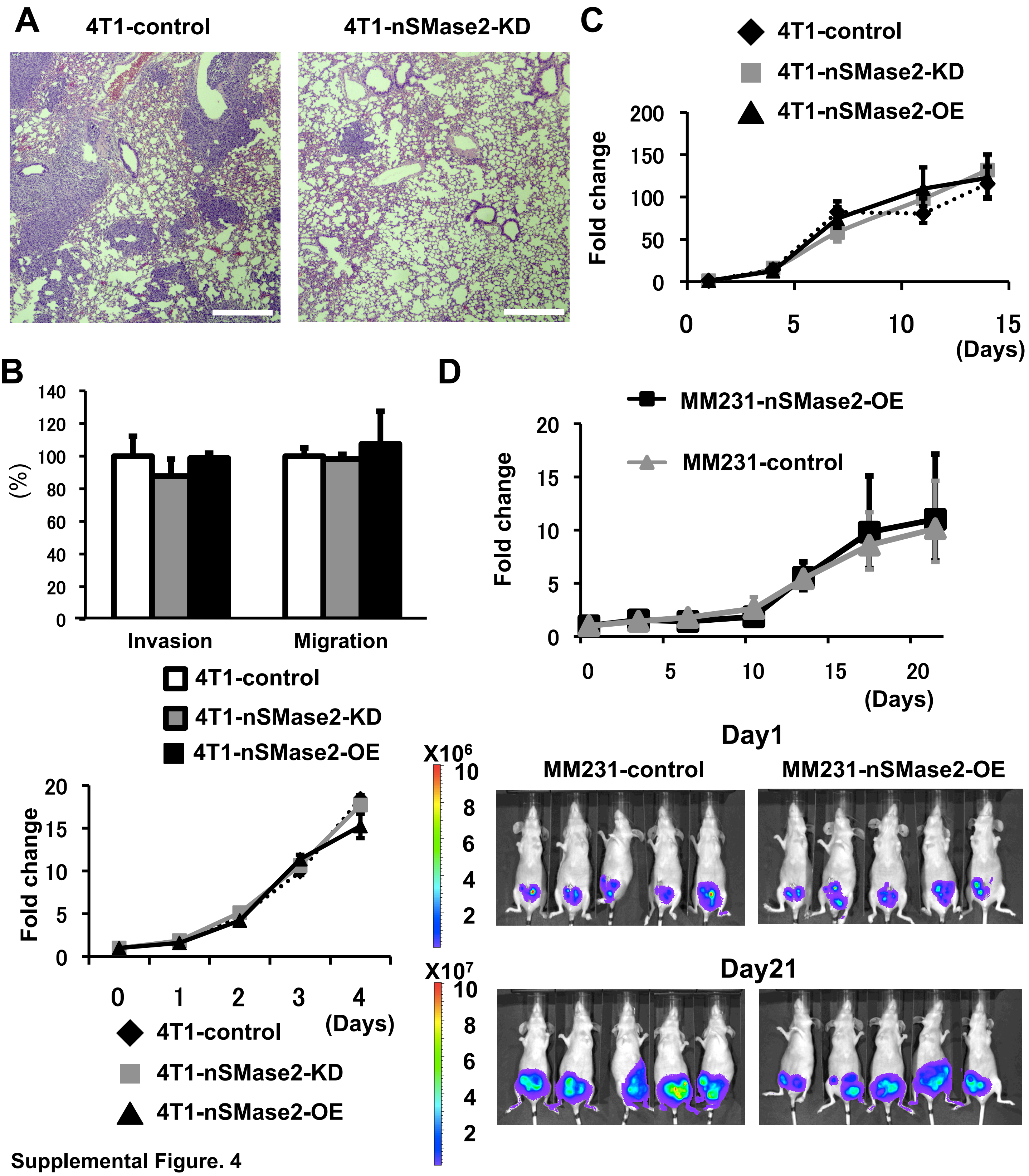

Supplemental Figure. 4

(A) Hematoxylin/eosin staining (H&E) of lung tissue isolated from parental 4T1-control cells or 4T1-nSMase2-KD cells. Scale bars, 400 μm for H&E. (B) In vitro migration, invasion (upper panel) and proliferation (lower panel) assays were performed using nSMase2-modified 4T1 cells and control cells. Each bar is presented as the mean S.E. (n=3). (C) Tumor growth of nSMase2-modified 4T1 cell lines was measured by IVIS system after the implantation of established cell lines in nude mice. Each bar is presented as the mean S.E. (n=5). (D) Tumor growth of parental, nSMase2-overexpressing MDA-MB-231-D3H2LN cell lines was measured by IVIS system after the implantation of established cell lines in nude mice. Each bar is presented as the mean S.E. (n=5).

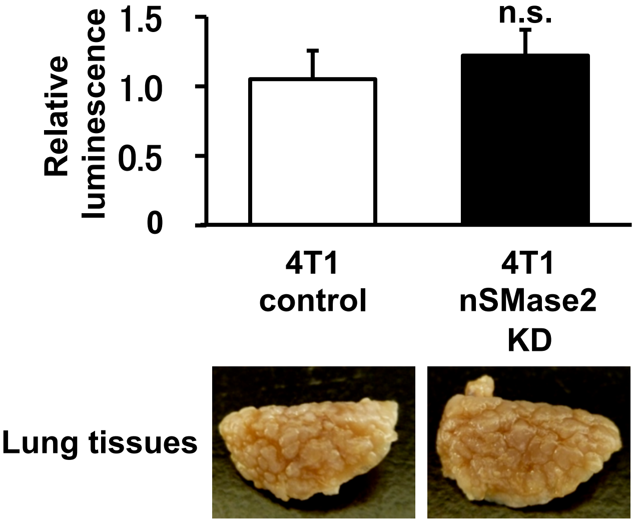

Supplemental Figure. 5
Bioluminescence quantification of lung metastasis following the intravenous injection of parental 4T1 cells or 4T1-nSMase2-KD cells. Each bar is presented as the mean S.E. (n=5). *n.s.* represents not significant.

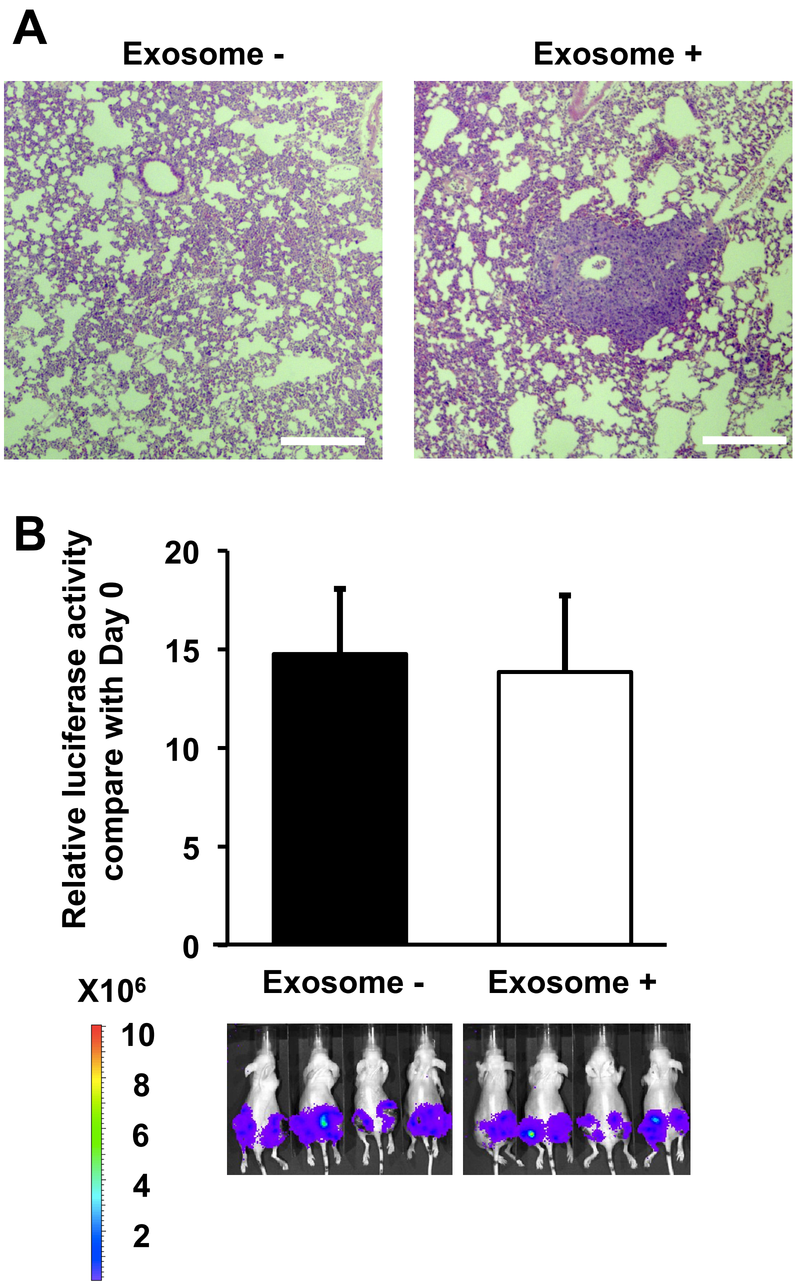

Supplemental Figure. 6
(A) Hematoxylin/eosin staining (H&E) of lung tissue isolated from 4T1-nSMase2-KD cells with or without the injection of exosomes. Scale bars, 200 μm for H&E. (B) Tumor growth of 4T1-nSMase2-KD cell lines in nude mice that did/did not receive exosome injections after the implantation of established cell lines in the nude mice. Each bar is presented as the mean S.E. (n=4).

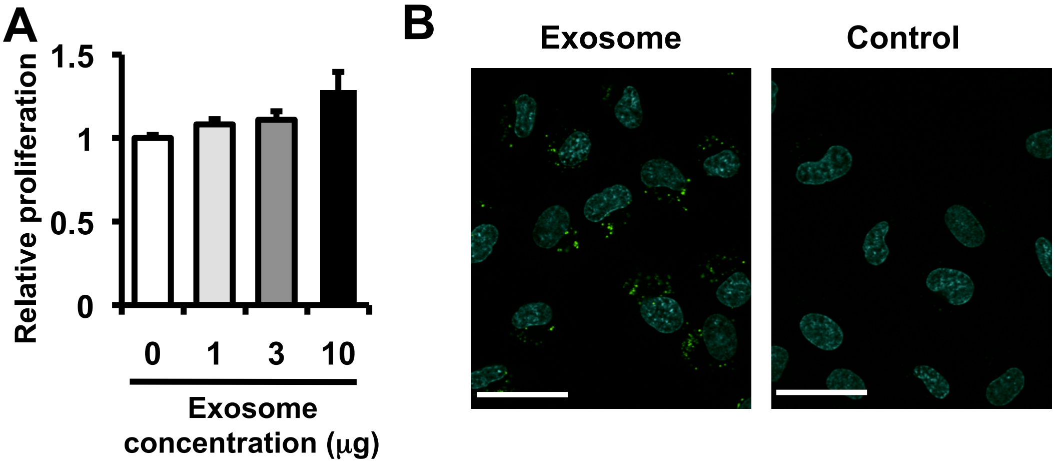

Supplemental Figure. 7
(A) The effect of exosome isolated from 4T1 cells against the cellular proliferation of HUVECs. (B) Fluorescence photos of HUVECs that had incorporated PKH67-loaded exosomes from 4T1 cells. HUVECs were incubated for 16 hr with PKH67-loaded exosomes. The size bar indicates 100 μm.

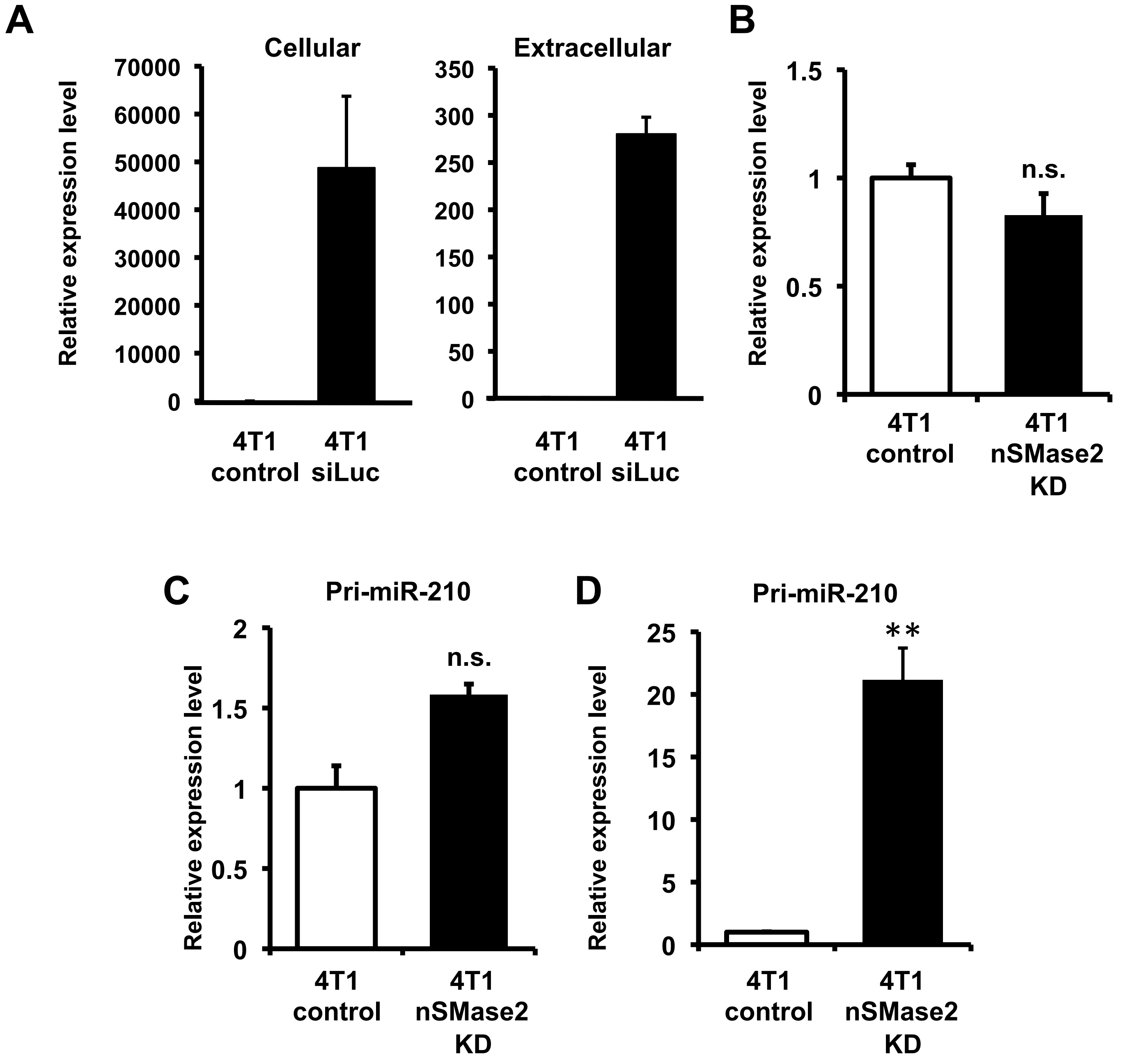

Supplemental Figure. 8

(A) Expression (left panel) and secretion of luciferase siRNA (right panel) was analyzed by qRT-PCR in parental 4T1-control cells and 4T1-siLuc cells. Each bar is presented as the mean S.E. (n=4). *n.s.* represents not significant. (B) The intracellular expression level of miR-210 in 4T1-control cells and 4T1-nSMase2-KD cells was measured by qRT-PCR. Each bar is presented as the mean S.E. (n=4). (C) Primary miR-210 expression in HUVECs was detected by qRT-PCR after co-cultured with parental 4T1 cells or 4T1-nSMase2-KD cells for 48 hr. β-actin was used as a control. Each bar is presented as the mean S.E. (n=3). *n.s.* represents not significant. (D) Primary miR-210 expression in HUVECs was detected by qRT-PCR after treatment with 30 mM of DFO for 48 hr. β-actin was used as a control. Each bar is presented as the mean S.E. (n=3). **, $p<0.005$, as compared with control cells.

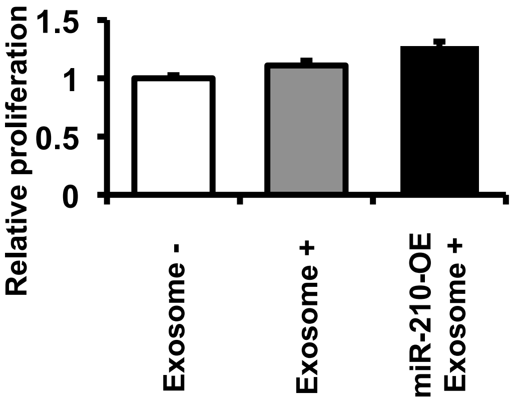

Supplemental Figure. 9
The effect of intact exsome and miR-210 enriched-exosome against the cellular proliferation of HUVECs.
